# Molecular insights into the mechanisms of a leaf color mutant in *Anoectochilus roxburghii* by gene mapping and transcriptome profiling based on PacBio Sequel II

**DOI:** 10.1038/s41598-023-50352-5

**Published:** 2023-12-20

**Authors:** Huiming Huang, Hui Zou, Hongting Lin, Yimin Dai, Jiangbo Lin

**Affiliations:** 1 Institute of Subtropical Agriculture, Fujian Academy of Agricultural Sciences, 1499 Jiulong Avenue, Zhangzhou, 363005 Fujian China; 2 Zhangzhou Fourth Municipal Hospital of Fujian Province, 41 Baiyun Village, Zhangzhou, 363100 Fujian China

**Keywords:** Biochemistry, Metabolomics

## Abstract

Plants with partial or complete loss of chlorophylls and other pigments are frequently occurring in nature but not commonly found. In the present study, we characterize a leaf color mutant ‘*arly01*’ with an albino stripe in the middle of the leaf, which is an uncommon ornamental trait in *Anoectochilus roxburghii*. The albino “mutant” middle portion and green “normal” leaf parts were observed by transmission electron microscopy (TEM), and their pigment contents were determined. The mutant portion exhibited underdevelopment of plastids and had reduced chlorophyll and other pigment (carotenoid, anthocyanin, and flavonoid) content compared to the normal portion. Meanwhile, comparative transcript analysis and metabolic pathways mapping showed that a total of 599 differentially expressed genes were mapped to 78 KEGG pathways, most of which were down-regulated in the mutant portion. The five most affected metabolic pathways were determined to be oxidative phosphorylation, photosynthesis system, carbon fixation & starch and sucrose metabolism, porphyrin and chlorophyll metabolism, and flavonoid biosynthesis. Our findings suggested that the mutant ‘*arly01*’ was a partial albinism of *A. roxburghii*, characterized by the underdevelopment of chloroplasts, low contents of photosynthetic and other color pigments, and a number of down-regulated genes and metabolites. With the emergence of ornamental *A. roxburghii* in southern China, ‘*arly01*’ could become a popular cultivar due to its unique aesthetics.

## Introduction

*Anoectochilus roxburghii* (Wall.) Lind. is one of the classical Chinese medicinal herbs belonging to the *Orchidaceae* family, whose wild populations are now endangered and extremely rare. In traditional Chinese medicine, *A. roxburghii* is used to clear heat (relieve the latent dryness-heat of the body), relieve coughing, relieve swelling, and detoxication^1^. Modern research shows that* A. roxburghii* has antitumor, antidiabetic, antihyperglycemic, antioxidant, immunostimulatory, and hepatoprotective activities^1–4^. Moreover, the *A. roxburghii* plant leaf is velvety and dark green in color with red-golden veins. These special and unique features make it an attractive ornamental plant. With the developments of cultivation techniques, the commercialization of *A. roxburghii* has quickly grown into a burgeoning industry, including facility cultivation, wild imitation planting, product processing, and sales^5–7^.

Leaf color mutations occur frequently in nature, but are rare in *A. roxburghii*. Our laboratory-bred variety ‘*arly01*’ is a leaf color mutant originating from the *A. roxburghii* wild type (WT). The discernible dissimilarity in appearance between ‘*arly01’* and *A. roxburghii* (WT) is that there is a light yellow-green stripe in the middle of the ‘*arly01’* leaf, which increases the aesthetic and commercial appeal of *A. roxburghii*.

There are numerous categories of leaf color mutant classifications^8^. Basically, it can be classified into the following phenotypes: albinism, yellowing, mottled or stripe, virescent, and the intermedium or mixed type of these phenotypes. Previous investigations have demonstrated that genetic changes in plant cells can directly or indirectly affect pigment synthesis, degradation, and proportions, leading to diverse types of leaf color mutations^9–11^. However, there have been few reports on the molecular mechanisms of *A. roxburghii* leaf color mutations, and the mechanism remains unclear.

The Pacific Biosciences (PacBio) Sequel II, providing circular consensus sequencing (CCS) and continuous long-read (CLR) sequencing modes, is the latest generation of a long-read sequencer platform. The PacBio Sequel II significantly improved the long-read sequencing and the discovery of candidate genes involved in biological processes, especially for plants without a reference genome (de novo sequencing) ^12,13^, such as *A. roxburghii* (improving the identified number of candidate genes associated with biological processes and their accuracy).

In the present study, we quantified and compared the pigment proportions in the leaves of the ‘*arly01*’ mutant. Subsequently, the PacBio Sequel II system was employed to conduct a transcriptome sequencing analysis (results were polished by the Illumina sequencing) of mixed RNA extracted from the different portions of ‘*arly01*’ (normal and mutated portions). After data analysis, specific genes and metabolic pathways associated with the leaf color mutation in *A. roxburghii* were identified and mapped. These transcriptome analyses have offered valuable insights into the molecular mechanisms underlying leaf color mutations in *A. roxburghii* and provided a valuable resource for improving and adapting ornamental plants.

## Materials and methods

### Plant materials

*Anoectochilus roxburghii* ‘Changtai’, wild type from Fujian, China, and the color mutant variety ‘*arly01*’, were grown in a greenhouse at the Institute of Subtropical Agriculture (ISA), Fujian Academy of Agricultural Sciences (FAAS, Zhangzhou, China, 117.738° E, 24.553° N). A voucher specimen of *A. roxburghii* ‘Changtai’ was also stored in the ISA of FAAS, voucher ID: ArCT20150321.

**Figure 1:**
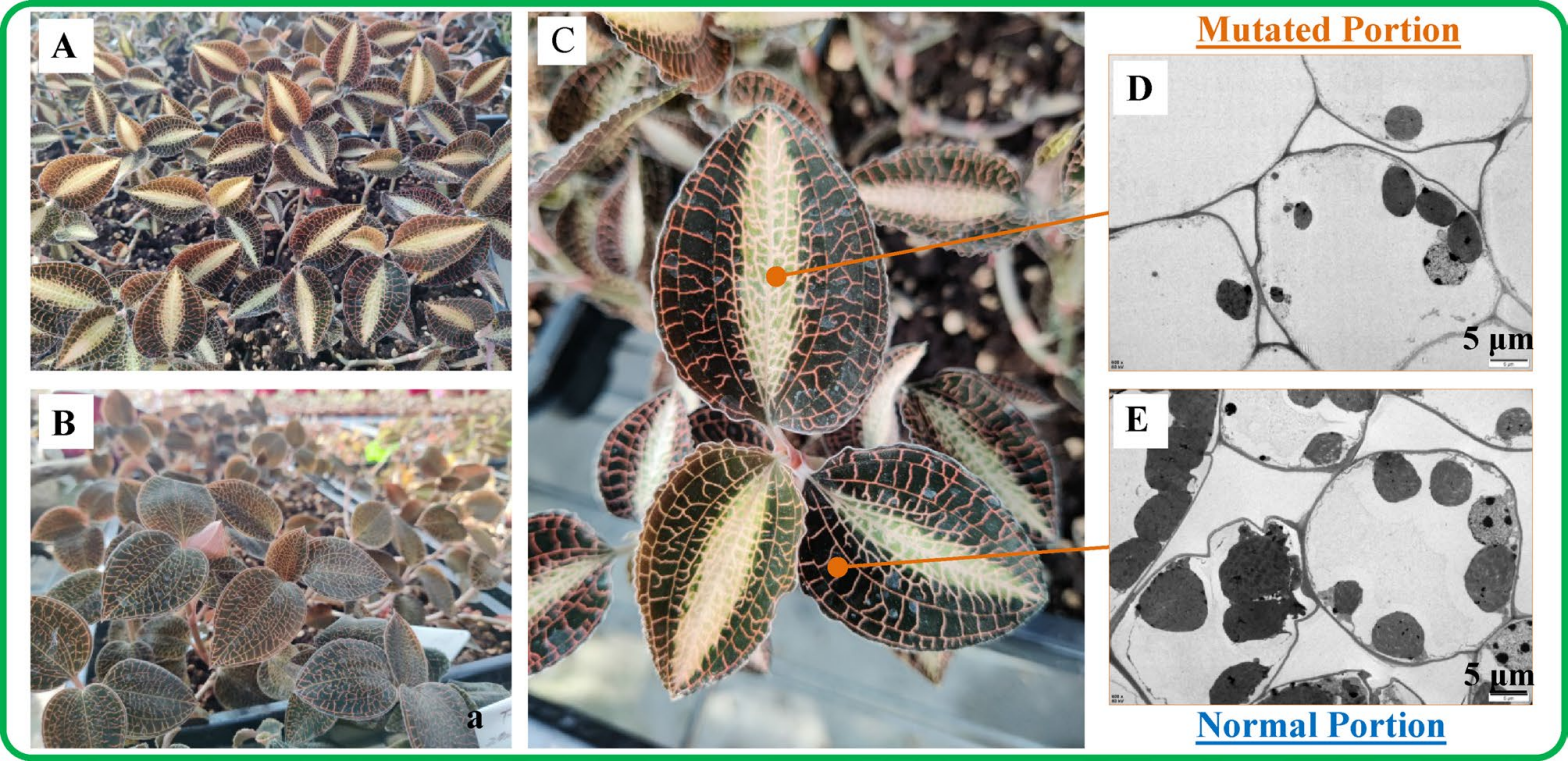
Phenotypes of the mutant ‘*arly01*’ (**A**) and wild type ‘Changtai’ (**B**) of *A. roxburghii* in the greenhouse. Overview of the chloroplast in the mutated portion (**D**) and normal portion (**E**) of the ‘*arly01*’ leaf (**C**) by transmission electron microscopic (TEM).

The ‘*arly01*’ was naturally mutated and got after 5 years of tissue culture and propagation using the ‘Changtai’ (WT) plant stems. Within the tissue culture and propagation process, axillary buds were used as propagules, which guaranteed their genetic backgrounds were consistent. Plant materials for experiments, ‘Changtai’ (WT) and ‘*arly01*’ (as shown in Fig. 1), were planted under 25 ± 2 °C with 3000–5000 Lux light intensity for 4 months. Experimental samples with three biological replicates were rapidly collected and frozen in liquid nitrogen and were immediately transferred and stored at − 80 °C for pigment extraction and subsequent RNA-seq analyses.

### Transmission electron microscopy (TEM) observations

The ‘*arly01*’ leaves were generally divided into two parts, the mutated portion (M, with yellow-green color) and the normal portion (N, with dark green color), as shown in Fig. 1C. The observation of the chloroplast ultrastructure was conducted using the M portion and N portion of the ‘*arly01*’ leaf. Leaves were first cut into 1 mm × 1 mm pieces and fixed in 2.5% (w/v) glutaraldehyde for 24 h at 4 °C, followed by 0.1 mol L^−1^ phosphate buffer (pH 7.4) washing for three times. Leaf samples were post-fixed in 1% (w/v) OsO_4_ at 4 °C for 2 h and washed with 0.1 mol L^−1^ phosphate buffer for three times. Samples were then dehydrated through a graded ethanol series (50%, 70%, 80%, 90%, 100%, and 100%, v/v), embedded in pure SPI-Pon 812 resin (Epon 812 substitute), and polymerized at 60 °C for 48 h. Ultrathin sections with 60–80 nm thickness were obtained using the Leica EM UC7 ultramicrotome (Leica, Germany). Samples were stained with 0.2% (w/v) aqueous uranyl acetate followed by lead citrate, for 15 min, respectively. The ultrastructure of leaf samples was examined using a Tecnai G2 20 TWIN TEM (FEI, USA).

### Pigment extraction and measurement

Approximately 0.2 g of M and N portions of leaf material were used to determine the chlorophyll and carotenoid contents. Briefly, fresh leaves were ground with clean quartz sands in 1 mL 80% (v/v) acetone (pre-cooled to 4 °C). The pasty was transferred to a 15 ml tube, and more acetone was added to a final volume of 10 mL. The tube was wrapped up in tin foil and put in darkness. The extraction process was continued till the pasty turned white. The absorption at 470, 646, and 663 nm was measured using an L5S UV–Vis spectrometry (INESA, China). The total chlorophyll and carotenoid content was calculated using the following Eqs. ^14^: ${C}_{a}=12.21{A}_{663}-2.81{A}_{646}$, ${C}_{b}=20.13{A}_{646}-5.03{A}_{663}$, ${C}_{x+c}=\frac{1000{A}_{470}-3.27{C}_{a}-104{C}_{b}}{229}$, where *C*_*a*_*, C*_*b*_*, C*_*x*+*c*_ represent the contents of chlorophyll a, chlorophyll b, and total carotenoids (xanthophylls and carotenes), respectively.

Approximately 0.1 g of leaf material was used to determine the anthocyanin content. Leaves were ground in 10 mL acidified methanol (1% HCl, v/v) and extracted twice in darkness at 25 °C for 2 h. The homogenates were centrifuged at 4 °C, 12,000 r/min for 15 min. The absorbance of the supernatant was measured at 530 and 657 nm. The anthocyanin content was calculated using the following formula: ${\text{C}}=\frac{A530-0.3A657\times V}{m}$, where V represents the volume of the extract (ml), and m represents the weight of the fresh sample^15^. Acidified methanol (1% HCl, v/v) was used as the blank control.

Fresh leaf material was first dried at 60 °C to consistent weight. Dry samples were ground to fine powder followed by adding 95% (v/v) ethanol and ultrasonic for 30 min to extract flavonoids. The homogenates were centrifuged at 4 °C, 12,000 r/min for 15 min. Total flavonoid contents were determined by the NaNO_2_-Al(NO_3_)_3_-NaOH method. Briefly, 1 mL of the extracts was added with 0.3 mL 5% (w/v) NaNO_2_ solutions and incubated for 5 min, followed by adding 0.3 mL 10% (w/v) Al (NO_3_)_3_ solutions for 6 min. Then 4 mL 4% (w/v) NaOH solution was added to the mixture, and the volume was adjusted to 10 mL with 70% (v/v) ethanol. After 15 min, the absorbance of the solution was measured at 510 nm. The total flavonoid content was determined using a standard curve with rutin (Aladdin, China) as the standard.

### RNA extraction, cDNA library construction, and de novo sequencing

The M and N portions of the leaves were ground into powder in liquid nitrogen, respectively. Total RNA extractions were done using the RNAprep Pure Plant Kit (DP441, Tiangen, China) following the manufacturer’s instructions. RNA integrity and quantity were assessed using the Agilent 2100 Bioanalyzer (Agilent Technologies, USA), making sure all the RNA integrity numbers (RIN) were at least 8.0. RNA concentration was measured using Nanodrop 2000 (Thermo Fisher Scientific, USA). To ensure obtaining enough RNA for sequencing, each RNA sample was mixed from five individual plant leaves. RNA extraction, DNA library construction, and transcriptome sequencing were completed by Biomarker Technologies Corporation (Beijing, China) using Illumina HiSeq4000 (next-generation sequencing, NGS) and Pacific Biosciences (PacBio) Sequel II (third-generation sequencing, TGS) platform. Quality checks of the cDNA libraries were done by Qubit (> 20 ng/μL), and Agilent 2100 (fragment range 250–390 bp, without impurity peaks).

### Transcriptome assembly, gene annotation and KEGG pathway mapping

To acquire high-quality clean reads for assembly and analysis, data from the two platforms were combined and analyzed^16^. Raw reads obtained from the NGS were used to polish errors in the long reads from the PacBio Sequel II platform, resulting in a more accurate and complete assembly of the transcriptome. The PacBio Sequel II transcriptome sequencing mainly includes the following three steps. First, raw reads were combined into circular consensus sequences (CCS) according to the adaptor. Next, full-length non-chimeric (FLNC) transcripts were determined by searching for the polyA tail signal, the 5′ and 3′ cDNA primers in CCS. Third, all full-length sequences from the same transcript and similar full-length sequences were clustered using the Iso-seq module in SMRT Link v7.0, and a consensus isoform sequence was obtained from each cluster. To get high-quality full-length transcripts an optimized CD-HIT^17,18^ (identity > 0.99) was set to remove redundancy.

Function annotation of assembled genes was performed using BLAST (version 2.2.26) based on public databases, including NR (NCBI non-redundant protein sequences), Swiss-Prot (A manually annotated and reviewed protein sequence database), GO (Gene Ontology), Pfam (Protein family), KOG/COG/eggNOG (Clusters of Orthologous Groups of proteins), and KEGG (Kyoto Encyclopedia of Genes and Genomes).

To identify the metabolic pathways genes and their biological functions in leaf color mutation of *A. roxburghii*, assembled unigenes were assigned to KEGG Automatic Annotation Server (KAAS, https://www.genome.jp/tools/kaas/) for ortholog assignment and pathway mapping based on sequence similarity.

### Differentially expressed genes (DEGs) analysis

Gene expression levels were estimated based on the FPKM (fragments per kilobase of transcript per million fragments mapped) method, as derived by the following equation:

$$FPKM = \frac{cDNA\;Fragments}{{Mapped\;Fragments\;\left( {Millions} \right) \times Transcript\;Length\left( {kb} \right)}}$$

Identification of the differentially expressed genes (DEGs) was performed with DESeq2^19^. DESeq2 provides statistical routines for determining differential expression in digital gene expression data using a model based on a negative binomial distribution. The resulting *p-values* were adjusted using Benjamini and Hochberg’s approach to control the false discovery rate (FDR). Genes with an FDR < 0.01 and |log_2_(fold change)|≥ 1 were assigned as differentially expressed. GO enrichment and KEGG pathway enrichment analysis were performed using GoSeq R and KOBAS^20^, respectively.

### Validation of DEGs by quantitative real‑time PCR

The RT-qPCR was performed using the SYBR®Green Premix Pro Taq HS qPCR Kit (No. AG11701, Accurate, China) in a Roche lightcycle®96 real-time PCR system (Roche, Germany), with 3 technical replicates. The qPCR volume was 20 μL in total, including 3 μL template cDNA, 1 μL forward primer and 1 μL reverse primer, 10 μL SYBR®Green Pro Taq HS Premix, and 5 μL RNase-free water. Six DEGs were selected for the validation of the transcript data. A housekeeper gene, *A. roxburghii β-Actin*, was used as the internal reference ^21^, which provided a basis for the relative quantification assays. The two-step cycling qPCR method was used in our experiment. Briefly, the denaturation was 95 °C for 30s, following 62 °C, 30s of annealing for 40 cycles. All primers used in this assay are listed in Table 1.

**Table 1:** Primers of DEGs for real-time fluorescent quantitative PCR.

Gene name	Forward primer (5′-3′)	Reverse primer (5′-3′)
*HemH*	AAAGCGGAGATGGAGGAGTG	ATTCCACAGGTCCAACTCGG
*ChlM*	CCAAGCAGCCCTATTCGTTC	CGAGCTGGACCTTATTGACG
*GLU*	AGGTGGGATGTCTTTGGGAG	CAAAACGCCCTGAAGCAACC
*bHLH*	CTCCATGCTCCTCTGATTG	ATGAGCACTATTTGTGGGTC
*YABBY*	CCAAGCCAGACATTCCTCAC	ACTTGGACCGCTACTGTTGG
*LOB*	ACGTGGCGAAACTCCTGAAC	TAGCCAACGCATCCGTAGAC
*β-Actin*	AGATGAGGCACAGTCCAAGA	GCTGGAACATTGAAGGTCTC

### Statistical analysis

The statistical analyses of pigment contents were conducted with the one-way ANOVA LSD test (*p*-value < 0.01) using the IBM SPSS Statistics (version 25) statistical software.

### Ethical approval

All materials in this study are comply with relevant institutional, national, and international guidelines, legislation, and sub-section ethical approval and consent to participate.

## Results

### Chloroplast observations

Ultrastructure of the plastids were observed in both portions (Fig. 1D,E) by transmission electron microscopy (TEM). The TEM images revealed that plastids in the M portion leaf tissues were underdeveloped compared to the chloroplasts in the N portion, and their number was lower, no ultrastructural changes were observed.

### Qualitative and quantitative pigments analyses

**Figure 2:**
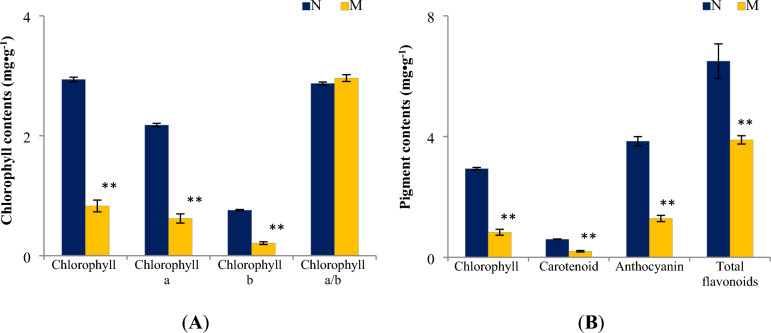
Chlorophyll (**A**) and other pigment contents (**B**) in the mutated portion (M) and the normal portion (N) of ‘*arly01*’ leaves (*p*-value < 0.01).

To determine the important pigments responsible for the leaf color mutation phenotype, pigment profile measurement was performed based on the M portion and N portion of ‘*arly01’* leaves. Results showed that color pigment profiles were significantly different between the M portion and the N portion. As shown in Fig. 2, pigments in the N portion, chlorophyll a, chlorophyll b, carotenoid, anthocyanin, and total flavonoids contents were 2.179 mg g^−1^, 0.758 mg g^−1^, 0.597 mg g^−1^, 3.844 mg g^−1^, and 6.497 mg g^−1^, respectively. Significant declines of 71.5%, 72.4%, 66.6%, 66.6%, and 40.1% were measured, respectively, in the M portion compared to the N portion. However, the value of (chlorophyll a/chlorophyll b) in the M portion was slightly higher than that of the N portion, which is not significant (*p*-value > 0.05).

### Overview of the transcriptome data and functional annotation

As shown in Supplementary Table S1, in the Illumina HiSeq sequencing, a total of 65.74 Gb clean data was obtained from the M and N portion leaves (three biological replicates for each portion). The dataset of each library ranged from 9.97 to 12.48 Gb. The GC contents of each sample ranged from 48.37 to 48.85%, and the percentages of base sequencing quality scores that reach 30 (referred to here as % ≥ Q30) of each library were greater than 94.73%.

A total of 537,890 polished CCS reads were obtained from the PacBio Sequel II sequencing platform (polished by the Illumina HiSeq sequencing data, all clean reads were merged), including 405,580 full-length non-chimeric (FLNC) reads which accounts for 75.40%. 130,098 consensus isoforms were obtained from each cluster by clustering of the FLNC reads, 130,057 of which were high-quality consistent sequences. CD-HIT^17,18^ (identity > 0.99) was set to remove the redundancy of the high-quality consistent sequences. Ultimately, the sequence and expression information of 82,215 genes was obtained for subsequent analysis. All these analysis algorithms ensure that our sequencing quality was sufficient for further analysis.

A total of 72,666 annotated unigenes were obtained based on the database searching against COG, GO, KEGG, KOG, Pfam, Swiss-Prot, EggNOG, and Nr, as shown in Supplementary Table S2.

### Functional analysis of DEGs between mutated and normal portions

**Figure 3:**
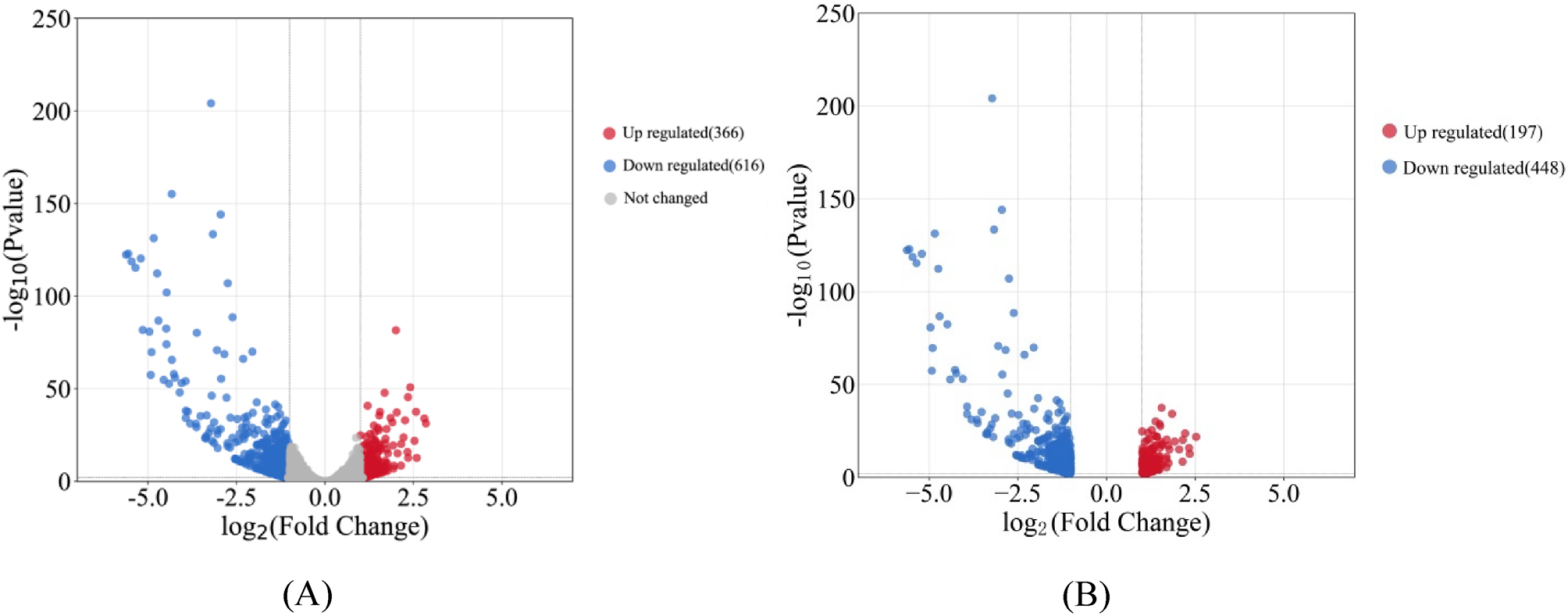
Volcano plots of gene expression profile between M portion versus N portion. Red points represent up-regulated DEGs. Blue points represent down-regulated DEGs. Gray points represent not changed genes. The identification thresholds were set as FDR < 0.01 and |log_2_(fold change)|≥ 1. (**A**) 982 identified DEGs, including 366 up-regulated and 616 down-regulated genes (M portion vs. N portion); (**B**) 645 GO annotated DEGs, including 197 up-regulated and 448 down-regulated genes (M portion vs. N portion). The top three abundant GO terms were metabolic process (387 DEGs), cellular process (298 DEGs), and single-organism process (239 DEGs).

**Figure 4:**
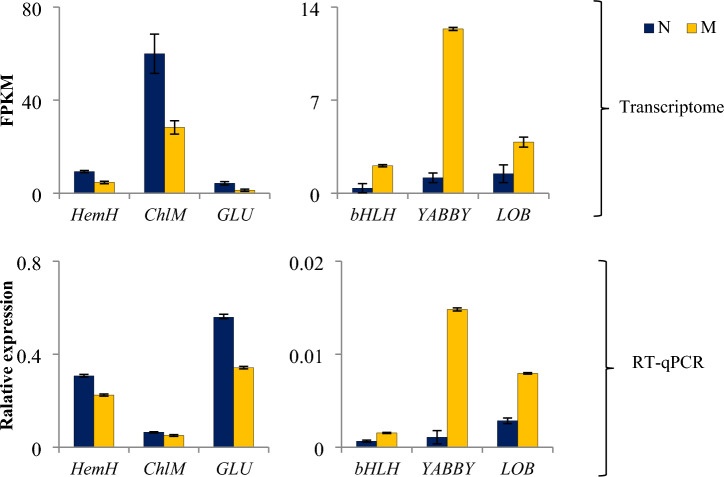
Comparison of the expression levels of six selected genes detected by transcriptome and RT-qPCR experiment. Results were presented as the mean of three repeated experiments.

FPKM method was used to evaluate the unigene expression levels. A total of 982 DEGs were identified, including 366 up-regulated and 616 down-regulated genes (M portion vs. N portion), as shown in Fig. 3. A heatmap of all the DEGs based on the FPKM values (normalized by the z-score method) showed that all the replicates exhibited similar expression patterns (Supplementary Figure S1). To further confirm these observations, six genes were selected for qRT-PCR validation (Fig. 4). The results of the qRT-PCR analysis were consistent with our transcriptome data and thus validated its reliability.

### GO and KEGG annotation analysis

**Figure 5:**
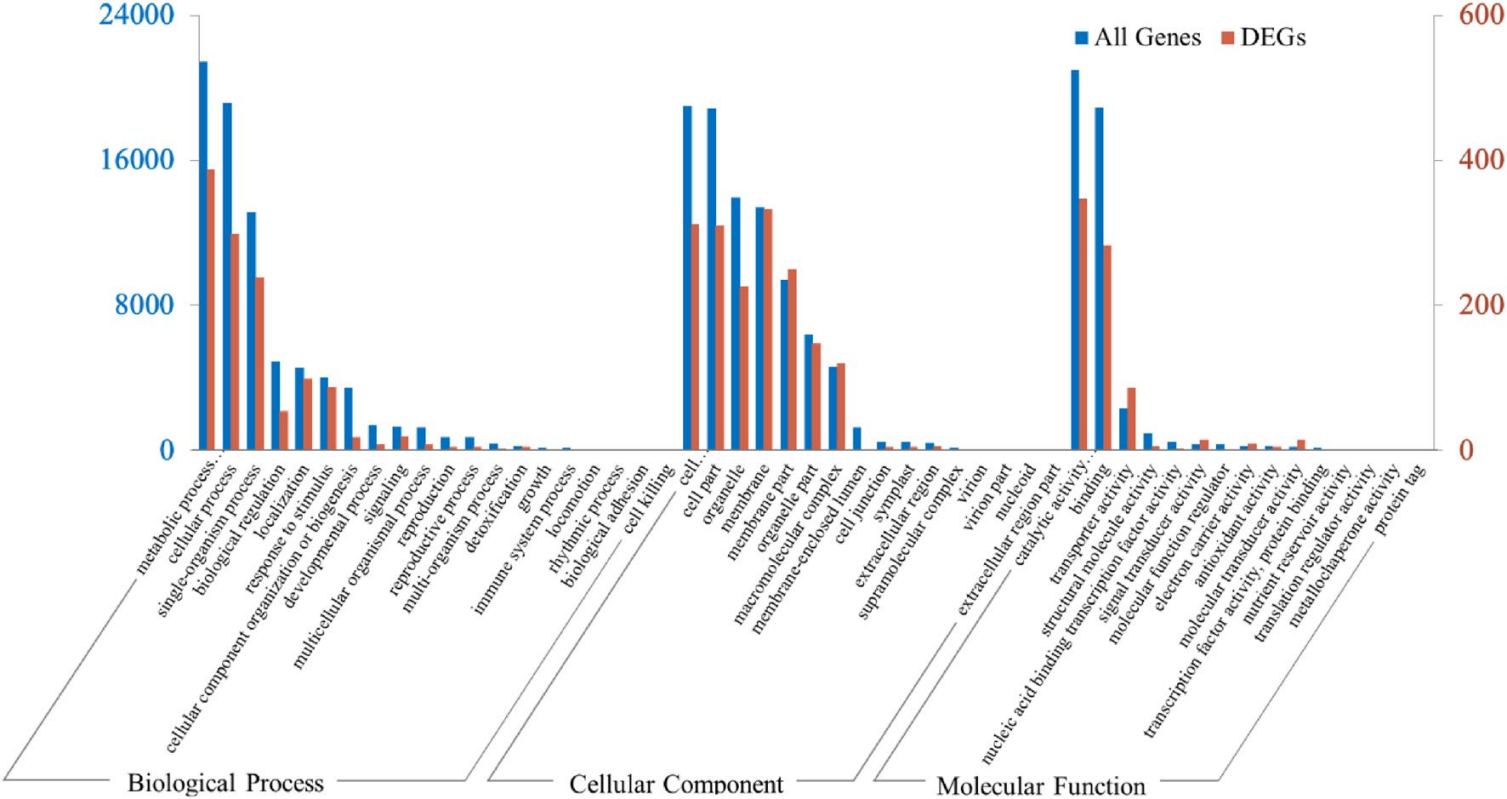
GO enrichment analysis of the genes of mutation portion vs. normal portion from ‘*arly01*’ leaves. A total of 40,206 unigenes and 645 DEGs were divided into three categories: biological process (BP), cellular component (CC), and molecular function (MF). Red columns represent the number of unigenes involved in the GO terms, blue columns represent the number of DEGs.

A total of 40,206 unigenes and 645 DEGs were assigned in GO annotation. GO enrichment analysis of all unigenes and DEGs were conducted to illustrate the distribution of genes involved in the following classifications: biological process (BP), cellular component (CC), and molecular function (MF), as shown in Fig. 5.

Within the biological process category, the top three abundant GO terms were metabolic process (387 DEGs, 60% accounted for all the 645 annotated DEGs), cellular process (298 DEGs, 46.20%), and single-organism process (239 DEGs, 37.05%). In the cellular component category, 333 and 250 DEGs (51.63%, 38.76%) were distributed in membrane and membrane part, 312 and 311 DEGs (48.37%, 48.21%) in cell and cell parts, 226 and 148 DEGs (35.03% and 22.95%) in organelle and organelle part. In terms of molecular function category, catalytic activity (347 DEGs, 53.80%), binding (283 DEGs, 43.87%), and transporter activity (87 DEGs, 13.49%) were the top three abundant GO terms.

High percentage of DEGs in these GO terms implied significant shifts or differences in the chemical and biological system between the N portion and M portion, such as in metabolite production, energy utilization, molecule transportation and movement, cell growth, signal transduction, and ligand-receptor interaction, etc. This was consistent with the results mentioned previously, such as the underdevelopment of plastids, decease of chlorophyll and other pigment contents and in the M portion.

**Figure 6:**
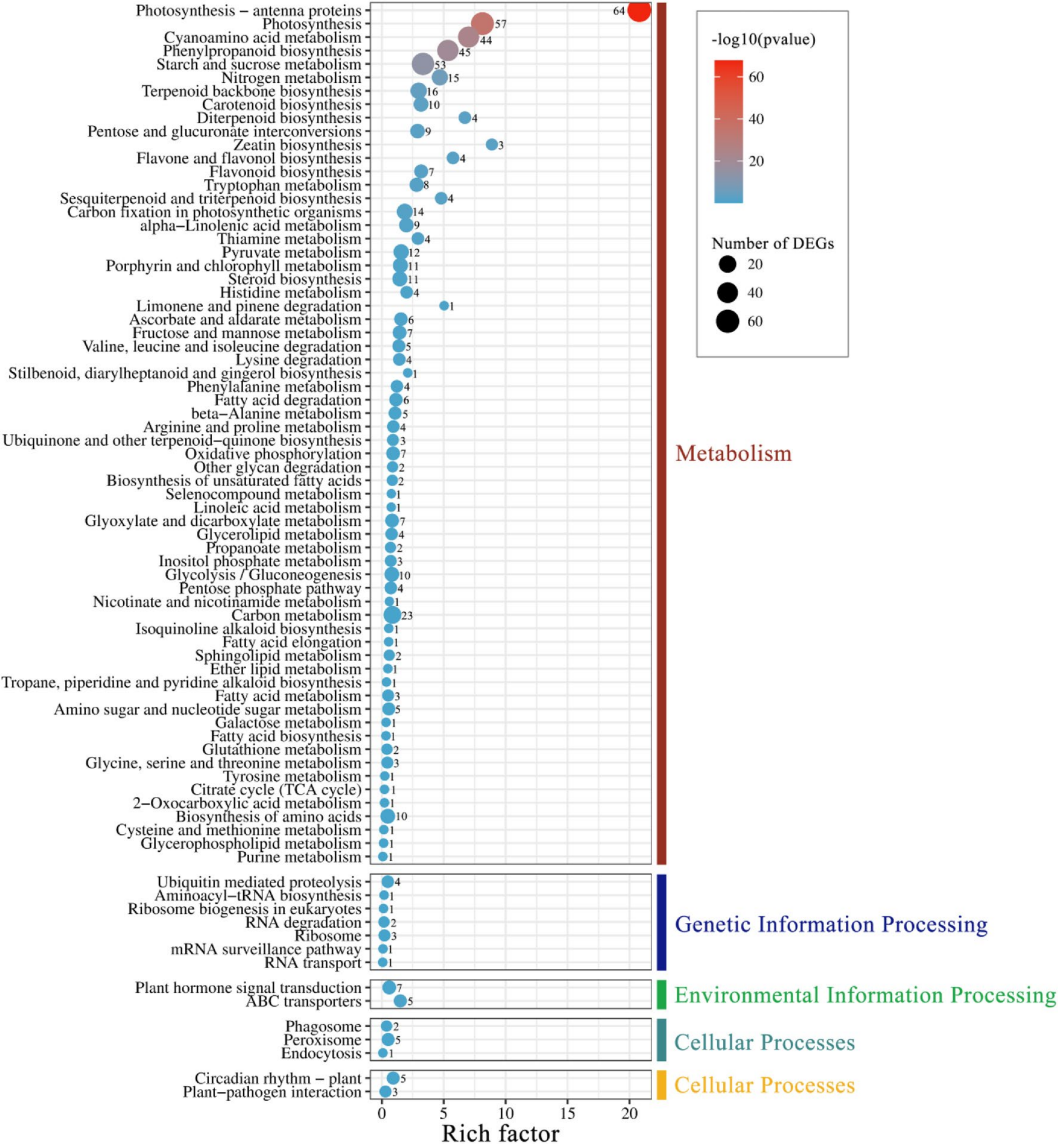
Enriched and classified KEGG pathways of the DEGs between the M portion versus N portion of the ‘*arly01*’ leaves. Color and size of the dots indicated the *p*-value and the number of DEGs (numbers noted next to the dots) mapped to a certain pathway, respectively. The rich factor (x-axis) is the ratio of the DEG number to the total gene number in the same pathway, represented as dots position. The pathways were further classified into five major groups: metabolism, genetic information processing, environmental information processing, cellular processes, and organismal systems.

To determine the significance of the mapped KEGG pathway and the DEGs enrichment, pathway significance enrichment analysis was conducted. A total of 599 DEGs were mapped to 78 KEGG pathways and were further classified into five classifications, including metabolism, genetic information processing, environmental information processing, cellular processes, and organismal systems. As shown in Fig. 6, the higher value of − log_10_ (*p*-value) for a certain pathway in our study indicated a higher significance of the pathway correlated to the conditions. Rich factors that represented the ratio of DEG numbers located in the same pathway were also calculated.

### Metabolic pathways and mapping

**Figure 7:**
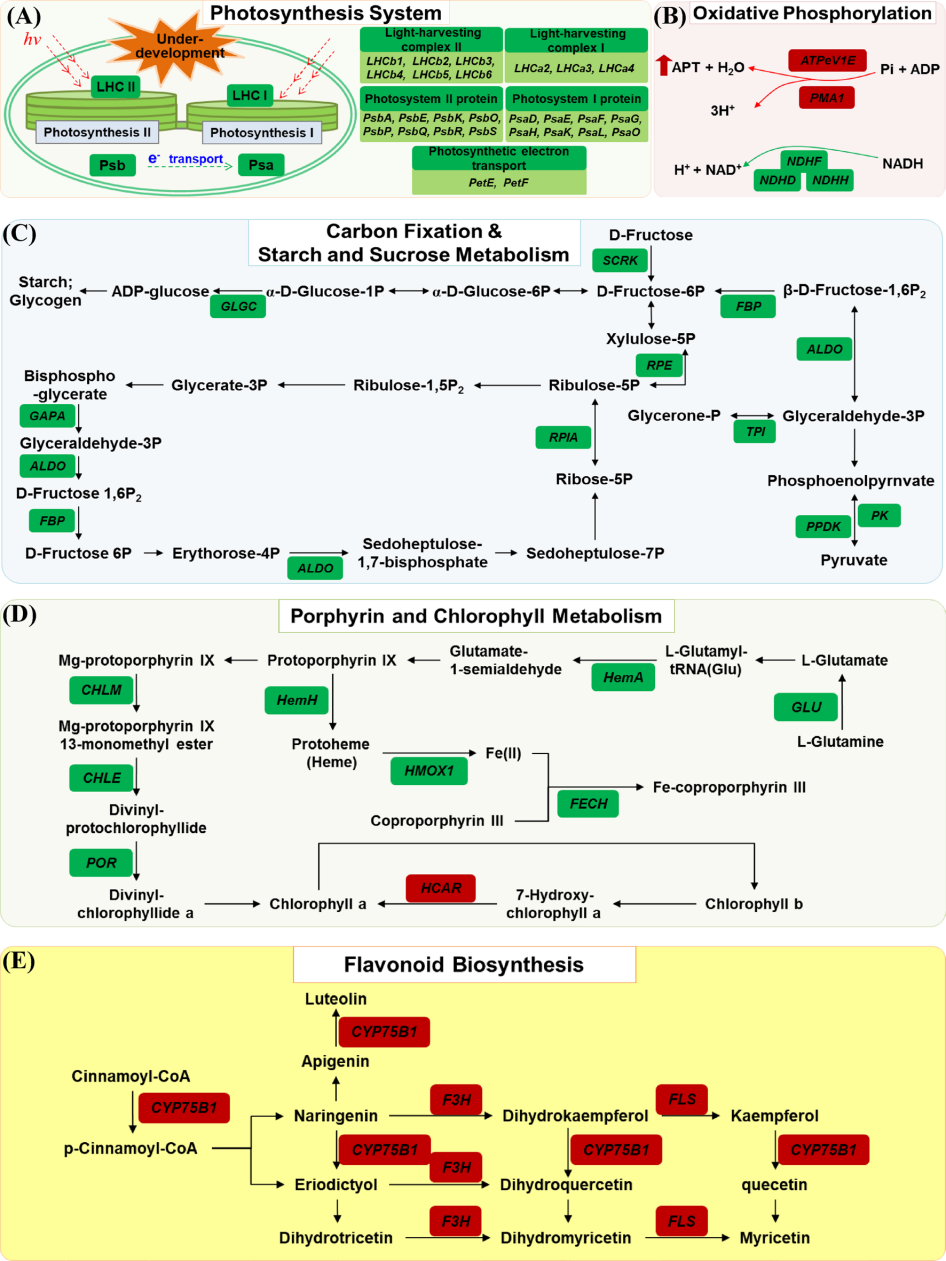
Overview of the differentially expressed genes (DEGs) that were involved in top-influenced metabolic pathways from the leaf color mutant ‘*arly01*’. Genes in red boxes and green boxes represented up-regulated genes and down-regulated genes, respectively. The metabolic processes include (**A**) photosynthesis system, (**B**) oxidative phosphorylation, (**C**) carbon fixation and starch and sucrose metabolism, (**D**) porphyrin and chlorophyll metabolism, and (**E**) flavonoid biosynthesis. Gene information is listed in Supplementary Table S3.

Based on the overall analysis of DEGs identification and KEGG classification, we picked the top 30 pathways and constructed five metabolic pathway maps influenced by the leaf color mutation, as shown in Fig. 7. DEGs involved in the pathways of oxidative phosphorylation (ko00190), photosynthesis system (ko00195 and ko00196), carbon fixation (ko00710) & starch and sucrose metabolism (ko00500), porphyrin and chlorophyll metabolism (ko00860), and flavonoid biosynthesis (ko00941) were sorted out (Supplementary Table S3).

Due to the underdevelopment of plastids, the photosynthesis antenna protein genes in light-harvesting complex I (includes chlorophyll a binding proteins of *LHCa2*, *LHCa3*, and *LHCa4*) and light-harvesting complex II (includes chlorophyll b binding protein of *LHCb1*, *LHCb2*, *LHCb3*, *LHCb4*, *LHCb5*, and *LHCb6*) were down-regulated**.** Eight genes of Photosystem I protein subunits (*PsaD*, *PsaE*, *PsaF*, *PsaG*, *PsaH*, *PsaK*, *PsaL*, and *PsaO*), and eight genes of Photosystem II protein subunits (*PsbA*, *PsbE*, *PsbK*, *PsbO*, *PsbP*, *PsbQ*, *PsbR*, and *PsbS*) were down-regulated. The *PetE* (plastocyanin), and *PetF* (ferredoxin) of photosynthetic electron transport genes were down-regulated (Fig. 7A). Influenced by this, in the oxidative phosphorylation pathway, the V-type H^+^-transporting ATPase subunit E(*ATPeV1E*) and H^+^-transporting ATPase(*PMA1*) were up-regulated, while three NAD(P)H-quinone oxidoreductase subunits genes (*NDHD*, *NDHF*, and *NDHH*) were down-regulated (Fig. 7B). A total of 10 down-regulated DEGs were identified in the carbon fixation & starch and sucrose metabolism pathway map, including *SCRK*, *GLGC*, *FBP*, *RPE*, *GAPA*, *ALDO*, *RPIA*, *TPI*, *PPDK*, and *PK* (Fig. 7C). Furthermore, nine genes of porphyrins and chlorophyll metabolisms such as *GLU*, *HemA*, *HemH*, *ChlM*, *ChlE*, *POR*, *HMOX1*, *FECH*, and *HCAR* were involved. Among the other down-regulated DEGs, the *HCAR* was the only up-regulated gene (Fig. 7D). Unlike the above-mentioned pathway, we identified three up-regulated DEGs that influenced many flavonoid biosynthesis in the flavonoid biosynthesis pathway, including *CYP75B1*, *F3H*, and *FLS* (Fig. 7E).

## Discussion

### Pigments and leaf color

The relationship between different pigments and leaf color is complex in plant biology. The pigment profiles between the M and N portions suggested that the mutation of ‘*arly01*’ leaves had significant impacts on the pigments and biochemical compositions.

Chlorophyll a (Chl a) and chlorophyll b (Chl b) are essential for photosynthesis, and their levels are crucial for determining leaf greenness^22^. The decline of 71.5% Chl a and 72.4% Chl b in the M portion significantly reduced the portion’s green color. Carotenoids^23^ (Car) can appear yellow, orange, or red; anthocyanins^24^ are responsible for red, purple, and blue; while flavonoids often appear yellow or white in plants^25^. Collectively, the decrease in photosynthetic pigments (chlorophylls and carotenoids) and color-related pigments (anthocyanins and flavonoids) resulting in a yellow-green stripe on the leaves (Fig. 1C).

### Photosynthesis

Chloroplast is the organelle where the plant photosynthesis process and energy conversion occur^26,27^. It is composed of three parts: chloroplast membrane, thylakoid and stroma. There are light-harvesting complexes I and II, photosystem I and II, and photosynthetic electron transfer systems located on the thylakoid membrane, together responding for light capture and energy transfer from light energy to produce ATP and NADH.

In plant cells^28,29^, the photosystem I is composed of 16 proteins (PsaA ~ X), the photosystem II is composed of 27 proteins (PsbA ~ 28), the photosynthetic electron transfer process is composed of 4 proteins (PetE ~ J), and the light-harvesting complexes I and II are composed of 5 (LHCa1 ~ 5) and 7 (LHCb1 ~ 7) proteins, respectively. The assembly of these proteins forms the PS I-LHC I supramolecular complex and PS II-LHC II supramolecular complex, with electron and energy transfers from PS II to PS I. The down-regulation of these genes (Fig. 7A) in the mutated portion of the ‘*arly01*’ leaf, is expected to reduce PS I-LHC I and PS II-LHC II supramolecular complexes, likely resulting in the underdevelopment of plastids, which was consistent with the results of electron microscope observation(Fig. 1D,E). The resulting lower efficiency of light absorption, energy transfer, and photosynthetic activity in the M portion consequently is expected to result in reduced production of glucose and other organic compounds.

Electrons from PS II are ultimately passed to PS I, where re-energization occurs with another photon of light energy^30^. Energized electrons from PSI are then used to reduce NAD^+^ to NADH, which is used in carbon fixation & starch and sucrose metabolism. However, in the current study, energy production genes were up-regulated, while the NADH oxidoreduction was down-regulated (Fig. 7B). A total of 10 genes were down-regulated in the carbon fixation & starch and sucrose metabolism pathway, as shown in Fig. 7C, which significantly reduced the storage of organic compounds in the mutated portion of ‘*arly01*’.

### Chlorophyll metabolism

Chlorophyll a, chlorophyll b, and carotenoids are the main photosynthetic pigments of higher plants, which also determine the color of plant leaves. Leaf color mutants are usually accompanied by changes of chlorophyll (Chl) and carotenoid levels ^31,32^. The *Ornamental crabapple* (*Malus sp.*) delayed-green leaf color mutant had lower Chl, Car, and flavonoid contents^33^. A study on the chlorophyll-deficient leaf color mutant of tree peony (*Paeonia suffruticosa*) showed that the mutant had lower pigment contents, but increased Chl a/b ratio and Car to Chl ratio^34^. In the present study, the content of Chl and Car in the mutated portion of ‘*arly01*’ was significantly decreased (*p*-value < 0.01), and the decreased ratio of Chl was higher than that of Car (*p*-value < 0.01). However, there was no significant difference found in the Chl a/b value between the M and the N portion, suggesting that the decrease of Chl a and Chl b was uniform.

As shown in Fig. 7D, the entire process of chlorophyll synthesis can be roughly categorized into three main steps^35^: the first step is the conversion of L-Glutamine into protoporphyrin IX (occurs in the cytoplasm), the second step is the insertion of magnesium ions into protoporphyrin IX (Mg-proto IX, completed in the middle of the chloroplast stroma), and the third step takes place in chloroplast membrane for the transformer process of Mg-proto IX to chlorophyll. Differential expression of any genes in the three steps leads to changes in enzyme function and activity that affect the whole chlorophyll synthesis process^36^, such as the accumulation of intermediates^37^, metabolism of chlorophyll^8^, and the distribution of chlorophyll pigment in plant cells^37,38^, which causes leaf color mutations, consequently.

In this study, the down-regulated expression of gene *GLU* and *HemA* (first step), *CHLM* and *CHLE* (second step), and *POR* (third step) reduced the efficiency of the Chl a and Chl b production. In addition, cross-conversion can happen between Chl a and Chl b in green plants, and we detected an up-regulated *HCAR* gene that could affect the efficiency of the transformation of Chl b to Chl a, possibly leading to the slight increase of chlorophyll a/b value (no significant difference) detected between the M portion and the N portion (Fig. 2).

### Flavonoid biosynthesis

Flavonoids play pivotal roles in plant development^39,40^, such as cell wall synthesis, pigmentation, pest resistance, and protecting plants from UV irradiation and oxidative stress. Flavonoids in *A. roxburghii* were well studied^41–43^. In the current study, three up-regulated genes were identified in the flavonoid biosynthesis pathway (Fig. 7E). However, the total flavonoid content of the M portion was significantly decreased (Fig. 2B). This paradox might be attributed to the decrease of photosynthesis efficiency, stimulating the production of plant secondary metabolites like flavonoids and phenolics^44^. However due to the lack of intermediate substances for flavonoid synthesis, which derived from the decreased photosynthesis and substances storage, the total flavonoid content was decreased^45^. Despite this, the *‘arly01’* mutant necessitates flavonoids in favor of leaf development and protection. Thus, a few flavonoid biosynthesis related genes were found up-regulated. Future efforts should be conducted to test this hypothesis and demonstrate the broad roles of flavonoids in *A. roxburghii*.

## Conclusions

In the present study, we have gained a comprehensive understanding of the leaf color mutation mechanism in the *A. roxburghii* variant, ‘*arly01*’ by using TEM, pigment profile analysis, comparative transcript analysis, and metabolic pathway mapping. Underdevelopment of plastids and significant declines of chlorophyll and other pigment contents were detected in the mutated portion. A series of negatively affected metabolic pathways were found and mapped, including the photosynthesis system, oxidative phosphorylation, carbon fixation & starch and sucrose metabolism, porphyrin and chlorophyll metabolism, and flavonoid biosynthesis. In conclusion, the variation of this mutant ‘*arly01*’ is characterized by the underdevelopment of plastids, low contents of photosynthetic and other color pigments, and several down-regulated genes and metabolites.

Further research can focus on strategies that control leaf color and improve functional ingredient products, such as deeper exploration of plastid gene mutations influencing leaf color^46^, breeding novel color variations in ornamental plants, and increasing flavonoid content in *A. roxburghii*.

### Supplementary Information

Supplementary Information.

## Data Availability

All materials and related data in this study are available upon request. The datasets generated during and/or analyzed during the current study are available in the NCBI Sequence Read Archive (SRA) repository, https://www.ncbi.nlm.nih.gov/bioproject/PRJNA973204.
