# Supplementary material for: Molecular insights into the mechanisms of a leaf color mutant in Anoectochilus roxburghii by gene mapping and transcriptome profiling based on PacBio Sequel II
Source: Sci Rep. 2023 Dec 20;13:22751. doi: 10.1038/s41598-023-50352-5 (PMC10733416; doi:10.1038/s41598-023-50352-5)
Supplement: Supplementary file 1 — Supplementary Information. [file 41598_2023_50352_MOESM1_ESM.docx]

**SUPPLEMENTARY MATERIAL**


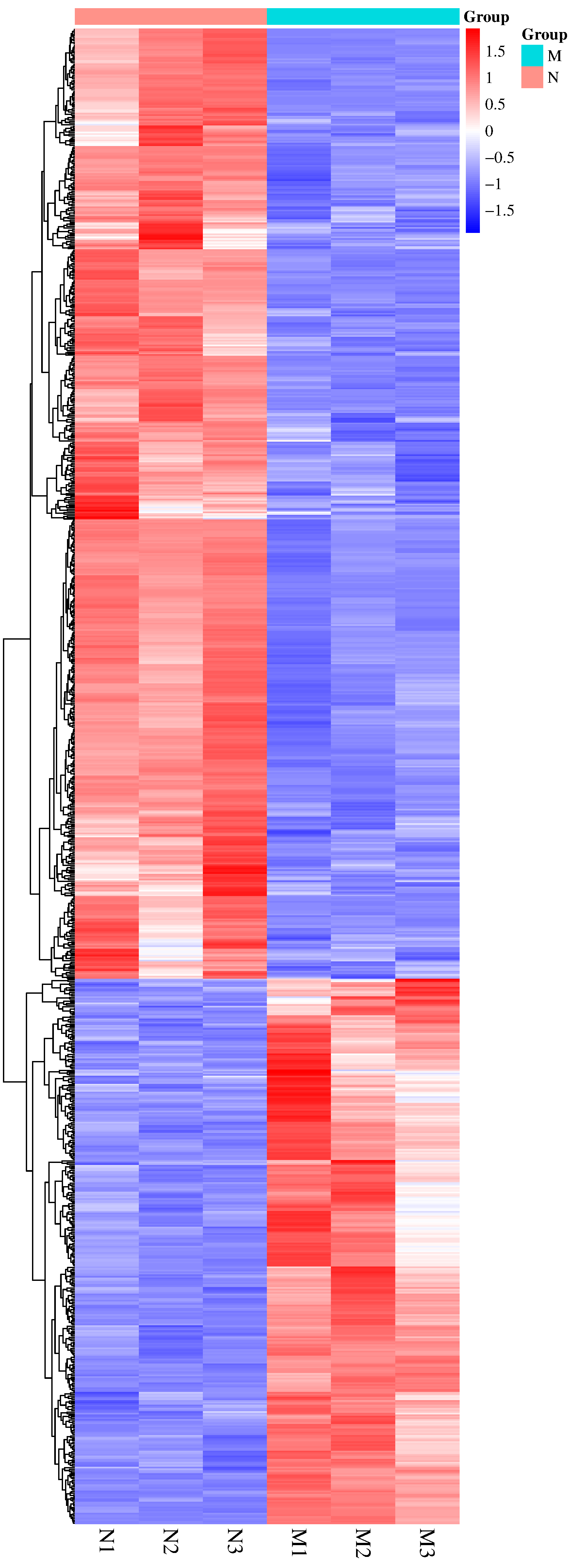


**Supplementary Figure S1.** Heatmap of all the DEGs based on the FPKM values (normalized by the z-score method). Heatmap was plotted by https://www.bioinformatics.com.cn, an online platform for data analysis and visualization.

**Supplementary Table S1.** Statistical table of the Illumina HiSeq sequencing data.

| Samples | Read Number | Base Number | GC Content | %≥Q30 |
| --- | --- | --- | --- | --- |
| M1 | 37,582,615 | 11,219,072,438 | 48.37 | 94.74 |
| M2 | 37,048,715 | 11,066,537,874 | 48.73 | 94.75 |
| M3 | 33,327,097 | 9,970,938,964 | 48.67 | 94.73 |
| N1 | 36,212,913 | 10,831,737,624 | 48.85 | 94.79 |
| N2 | 33,983,223 | 10,169,310,904 | 48.83 | 94.78 |
| N3 | 41,747,979 | 12,480,318,120 | 48.79 | 94.78 |

**Supplementary Table S2.** Statistics results of unigenes annotated in public databases.

| Databases | Number of transcript sequences | Percentage(%) |
| --- | --- | --- |
| COG | 27,652 | 36.12 |
| GO | 40,206 | 52.51 |
| KEGG | 30,839 | 40.28 |
| KOG | 46,175 | 60.31 |
| Pfam | 51,881 | 67.76 |
| Swiss-Prot | 49,696 | 64.91 |
| EggNOG | 69,069 | 94.33 |
| Nr | 72,219 | 94.91 |
| All | 72,666 |  |

**Supplementary Table S3.** DEGs involved in the TOP metabolic pathways of *arly01*.

| KEGG Pathway | Enzyme | Regulation | Annotation |
| --- | --- | --- | --- |
| Photosynthesis-antenna proteins (ko00196) | LhcA2 | Down-regulated | Light-harvesting complex I chlorophyll a/b binding protein 2 |
|  | LhcA3 | Down-regulated | Light-harvesting complex I chlorophyll a/b binding protein 3 |
|  | LhcA4 | Down-regulated | Light-harvesting complex I chlorophyll a/b binding protein 4 |
|  | LhcB1 | Down-regulated | Light-harvesting complex II chlorophyll a/b binding protein 1 |
|  | LhcB2 | Down-regulated | Light-harvesting complex II chlorophyll a/b binding protein 2 |
|  | LhcB3 | Down-regulated | Light-harvesting complex II chlorophyll a/b binding protein 3 |
|  | LhcB4 | Down-regulated | Light-harvesting complex II chlorophyll a/b binding protein 4 |
|  | LhcB5 | Down-regulated | Light-harvesting complex II chlorophyll a/b binding protein 5 |
|  | LhcB6 | Down-regulated | Light-harvesting complex II chlorophyll a/b binding protein 6 |
| Photosynthesis (ko00195) | PsbA | Down-regulated | Photosystem II P680 reaction center D1 protein |
|  | PsbE | Down-regulated | Photosystem II cytochrome b559 subunit alpha |
|  | PsbK | Down-regulated | Photosystem II PsbK protein |
|  | PsbO | Down-regulated | Photosystem II oxygen-evolving enhancer protein 1 |
|  | PsbP | Down-regulated | Photosystem II oxygen-evolving enhancer protein 2 |
|  | PsbQ | Down-regulated | Photosystem II oxygen-evolving enhancer protein 3 |
|  | PsbR | Down-regulated | Photosystem II 10kDa protein |
|  | PsbS | Down-regulated | Photosystem II 22kDa protein |
|  | PsaD | Down-regulated | Photosystem I subunit II |
|  | PsaE | Down-regulated | Photosystem I subunit IV |
|  | PsaF | Down-regulated | Photosystem I subunit III |
|  | PsaG | Down-regulated | Photosystem I subunit V |
|  | PsaH | Down-regulated | Photosystem I subunit VI |
|  | PsaK | Down-regulated | Photosystem I subunit X |
|  | PsaL | Down-regulated | Photosystem I subunit XI |
|  | PsaO | Down-regulated | Photosystem I subunit PsaO |
|  | PetA | Down-regulated | Apo cytochrome f |
|  | PetE | Down-regulated | Plastocyanin |
|  | PetF | Down-regulated | Ferredoxin |
|  | ATPF1A | Down-regulated | F-type H+/Na+-transporting ATPase subunit alpha |
| Porphyrin and chlorophyll metabolism (ko00860) | HemA | Down-regulated | Glutamyl-tRNA reductase |
|  | HemH | Down-regulated | Protoporphyrin/coproporphyrin ferrochelatase |
|  | ChlM | Down-regulated | Magnesium-protoporphyrin O-methyltransferase |
|  | HMOX1 | Down-regulated | Heme oxygenase 1 |
|  | CRD | Down-regulated | Magnesium-protoporphyrin IX monomethyl ester (oxidative) cyclase |
|  | POR | Down-regulated | Protochlorophyllide reductase |
|  | HCAR | Up-regulated | 7-hydroxymethyl chlorophyll a reductase |
| Oxidative phosphorylation (ko00190) | ATPeV1E | Up-regulated | V-type H+-transporting ATPase subunit E |
|  | PMA1 | Up-regulated | H+-transporting ATPase |
|  | NDHD | Down-regulated | NAD(P)H-quinone oxidoreductase subunit 4 |
|  | NDHF | Down-regulated | NAD(P)H-quinone oxidoreductase subunit 5 |
|  | NDHH | Down-regulated | NAD(P)H-quinone oxidoreductase subunit H |
| Carbon fixation (ko00710) & Starch and sucrose metabolism (ko00500) | SCRK | Down-regulated | Fructokinase |
|  | GLGC | Down-regulated | Glucose-1-phosphate adenylyltransferase |
|  | GAPA | Down-regulated | Glyceraldehyde 3-phosphate dehydrogenase (phosphorylating) |
|  | ALDO | Down-regulated | Alditol oxidase |
|  | FBP | Down-regulated | Fructose-1,6-bisphosphatase I |
|  | RPIA | Down-regulated | Ribose 5-phosphate isomerase a |
|  | RPE | Down-regulated | Ribulose-phosphate 3-epimerase |
|  | TPI | Down-regulated | Triosephosphate isomerase |
|  | PPDK | Down-regulated | Pyruvate, orthophosphate dikinase |
|  | PK | Down-regulated | Pyruvate kinase |
| Porphyrin and chlorophyll metabolism (ko00860) | GLU | Down-regulated | Glutamate synthase (ferredoxin) |
|  | HemA | Down-regulated | Glutamyl-trna reductase |
|  | CHLM | Down-regulated | Magnesium-protoporphyrin o-methyltransferase |
|  | CHLE | Down-regulated | Magnesium-protoporphyrin ix monomethyl ester (oxidative) cyclase |
|  | POR | Down-regulated | Protochlorophyllide reductase |
|  | HemH | Down-regulated | Protoporphyrin/coproporphyrin ferrochelatase |
|  | HMOX1 | Down-regulated | Heme oxygenase 1 |
|  | FECH | Down-regulated | Protoporphyrin/coproporphyrin ferrochelatase |
|  | HCAR | Up-regulated | 7-hydroxymethyl chlorophyll a reductase |
| Flavonoid biosynthesis(ko00941) | CYP75B1 | Up-regulated | Flavonoid 3'-monooxygenase |
|  | F3H | Up-regulated | Naringenin 3-dioxygenase |
|  | FLS | Up-regulated | Flavonol synthase |
